# Renal vein thrombosis after COVID‐19: A case report

**DOI:** 10.1002/ccr3.6778

**Published:** 2022-12-26

**Authors:** Hamid Janbazi Roudsari, Mohammad Negaresh, Vida Shirzadeh, Bahman Mohammadzadeh Germi, Arezoo Mirzaei

**Affiliations:** ^1^ Internal Medicine, Emam Khomeini Hospital Ardabil University of Medical Sciences Ardabil Iran; ^2^ Faculty of Medicine, Emam Khomeini Hospital Ardabil University of Medical Sciences Ardabil Iran; ^3^ Department of Radiology, Imam Khomeini Hospital Ardabil University of Medical Sciences Ardabil Iran

**Keywords:** angiotensin‐converting enzyme 2, COVID‐19, hypercoagulopathy, renal vein thrombosis

## Abstract

Severe cases of coronavirus disease 2019 (COVID‐19), caused by severe acute respiratory syndrome‐Coronavirus‐2, can lead to pneumonia or acute respiratory distress syndrome. Non‐respiratory manifestations of COVID‐19 include venous and arterial thrombosis. The disease can affect all organs and even the kidneys and lead to renal vein thrombosis where renal veins or their branches become thrombotic leading to symptoms such as flank pain, hematuria, or acute kidney damage. In this study, a case of renal vein thrombosis after COVID‐19 is introduced and the causes and complications of this disease are analyzed.

## Abstract 2

Hematuria and urinary symptoms in patients with COVID‐19, especially in patients who have risk factors for thrombosis, suggest the possibility of renal vein thrombosis, which requires the necessary measures to be taken to treat and prevent its thromboembolic complications.

## 1 INTRODUCTION

In December 2019, a sudden outbreak of pneumonia caused by severe acute respiratory syndrome‐coronavirus‐2 (SARS‐CoV‐2) occurred in Wuhan, China. Later called coronavirus disease 2019 (COVID‐19), the disease spread rapidly worldwide and became a pandemic.^1^ Common clinical manifestations of COVID‐19 are fever, cough, shortness of breath, fatigue, headache, hemoptysis, loss of taste or smell, and gastrointestinal manifestations such as nausea, vomiting, anorexia, diarrhea, and abdominal pain.^2^ The virus detects angiotensin‐converting enzyme 2 (ACE‐2) as its receptor and binds to it. ACE‐2 exists in almost all human tissues including endothelial cells of arteries, small and large veins, heart, alveolar epithelial cells type 1 and 2 in lungs, nasal mucosa, mouth, nasopharynx, kidney, testis, and brain.^3^ Many reports suggest that the introduction of SARS‐CoV‐2 is associated with decreased ACE‐2 activity and that ACE‐2 acts as a negative regulator of the renin‐angiotensin‐aldosterone (RAAS) system in critical situations. RAAS is thus boosted in COVID‐19 patients and causes oxidative stress damage to the vascular endothelium.^4^ In general, endothelial damage is associated with the pathophysiology of COVID‐19. Activation of von Willebrand factor (VWF), complement overactivation, excessive neutrophil extracellular traps (NETs) formation, and mitogen‐activated protein kinases may also play a role in COVID‐19‐related coagulation. Dysregulated innate immune response and subsequent cytokine storms would also lead to the activation of various “immunothrombotic” pathways and blood coagulation. COVID‐19‐related coagulation affects different organs including pulmonary arteries, lower limbs, spleen, heart, brain, and kidneys.^3^ Renal vein thrombosis (RVT) is a condition in which thrombosis occurs in the renal veins or their branches. Acute RVT is often due to trauma, severe dehydration, hypercoagulability, or nephrotic syndrome. RVT is rare and occurs most often in adults with nephrotic syndrome and newborns with low body fluid volume or inherited thrombophilia. While it may present with symptoms such as flank pain, hematuria, or acute kidney injury, it may also remain asymptomatic until it leads to pulmonary embolism (PE) or accidentally diagnosed by imaging studies.^5^

## 2 CASE PRESENTATION

**FIGURE 1:**
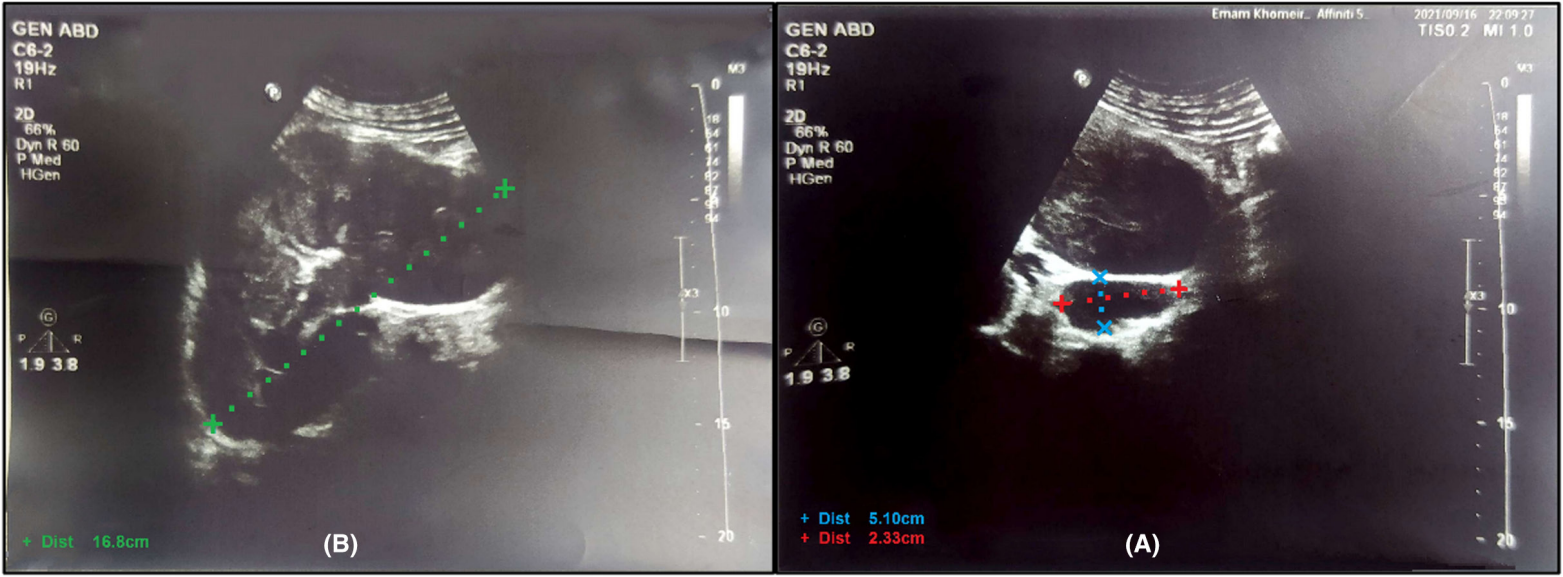
Color Doppler ultrasound of the left kidney. (A) Renal vein thrombosis in the left renal hilum measuring 51 × 23 mm (blue and red dotted lines) (B) Left kidney swollen and larger than normal with a longitudinal diameter of 168 mm (green dotted line)

A 49‐year‐old male patient presented to the hospital emergency department with left flank pain and hematuria. The patient's flank pain, which had started gradually from a week earlier, was intermittent and did not spread anywhere, but had intensified over the past 2 days. He also complained of hematuria along with flank pain occurring once or twice a day and manifested as voiding of blood clots in his urine. Symptoms of decreased urinary output, fever, chills, nausea, vomiting, diarrhea, and dysuria were not mentioned. He had a past history of extensive deep vein thrombosis (DVT) in the lower right limb about 5 years earlier, and as he did not have any provoking factors, thrombophilic tests was checked and showed negative results (antithrombin III, factor V Leiden, lupus anticoagulant, and anti‐cardiolipin IgG and IgM). Therefore, he received 7.5 mg daily warfarin tablets for 5 years. Six months earlier, he decided to discontinue treatment and take Aspirin 80 mg tablet daily without medical consultation. The patient also reported a history of COVID‐19 about 3 months earlier. He did not report a history of prolonged travel, surgery, cancer, trauma, or bedridden state. His sister had a history of DVT and died of pulmonary thromboembolism due to lack of cooperation in treatment process. On examination, the patient was conscious and oriented and his vital signs were blood pressure 120/80 mmHg, heart rate 126 beats/min, respiratory rate 19 breaths/min, saturations 96% on room air, and body temperature 36.5°C. On physical examination, the heart and lungs were normal and fatty abdominal distension. No bruit was heard in the renal artery pathway. Left lower quadrant and costovertebral angle tenderness was detected during abdominal examination. On limb examination, there was no swelling and both sides had similar sizes and strong symmetrical pulses. He was referred to the hospital with a color doppler ultrasound examination of kidneys which showed normal arterial flow velocity and pattern in intrarenal and main interlobar arteries in the right kidney. The size and parenchymal thickness of the right kidney were 129 and 16 mm, respectively. No pathological complication was observed in the right intrarenal and main renal veins. The left kidney was edematous and swollen leading to the loss of corticomedullary differentiation. The size and parenchymal thickness of the left kidney were 168 and 25 mm, respectively. A filling defect was evident in the left renal hilum, suggesting a thrombus measuring 51 × 23 mm in the main renal vein (Figure 1). Examination of the renal vein at the periphery of the inferior vena cava (IVC) and its distal portion also showed extensive thrombosis reaching the site where the vein drains into the IVC (70 mm in diameter) without evidence of thrombotic spread to the IVC. IVC had a normal flow. The resistance index of the interlobar arteries of the left kidney was at the upper normal limit, and left renal arterial flow was normal. Clear recanalization in the left renal vein was not appraisable.

**FIGURE 2:**
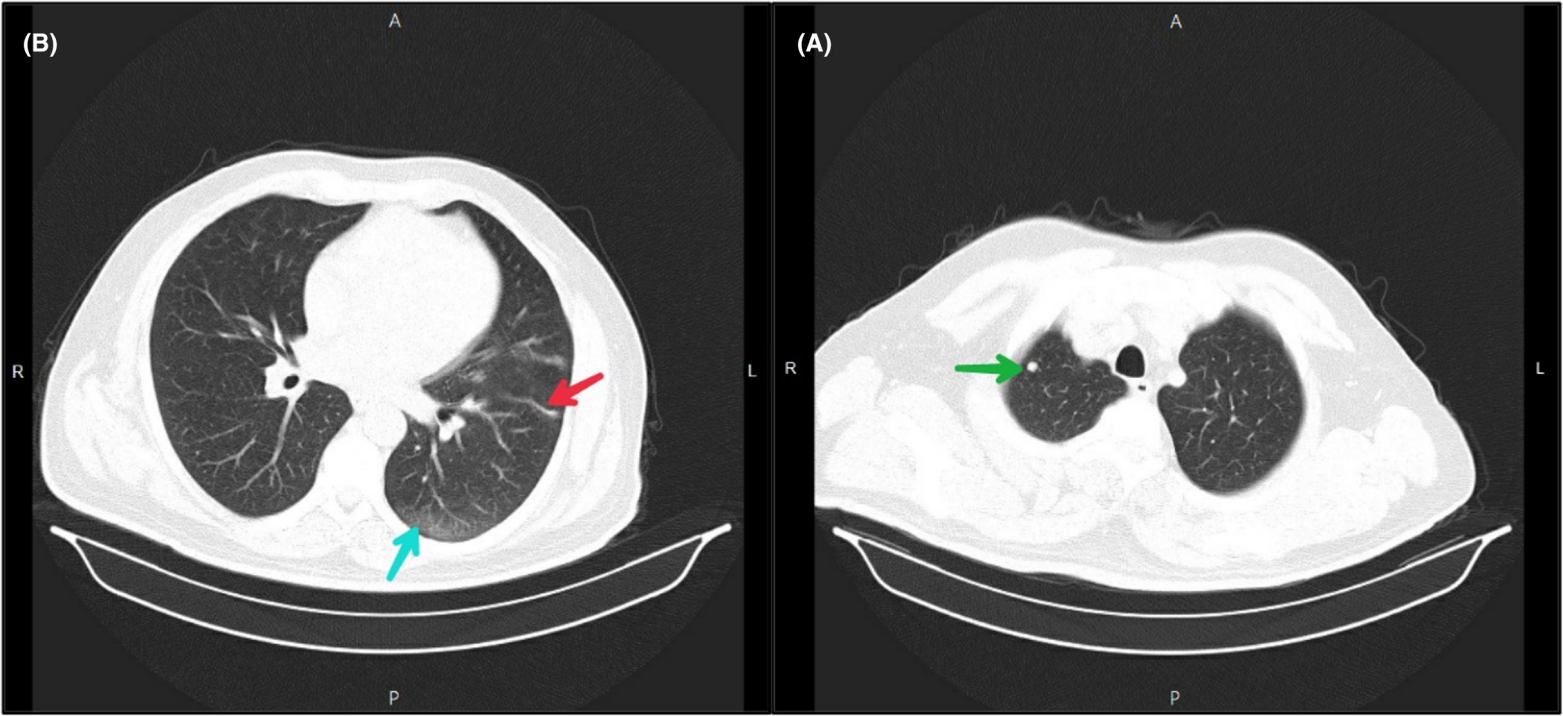
Parenchymal view of the spiral chest CT scan at admission. (A) Nodule with sharp edges in RUL (green arrow); (B) Peripheral ground glass opacity (blue arrow) and atelectatic band in the left lung parenchyma (red arrow)

At admission, a spiral chest CT scan without contrast due to the COVID‐19 pandemic along with relevant tests was performed. In lung imaging, the heart had a normal size. A 5 mm nodule with a sharp edge and without calcification component a similar nodule, 6.5 mm in diameter, in the lateral segment of its right middle lobe and a 10 mm nodule in the left lower lobe was observed in the right upper lobe. A number of atelectatic bands were evident in both lung parenchyma. Due to the recent viral pneumonia, several focal alveolar subsegmental and peripheral ground‐glass opacities were evident in the lung parenchyma (Figure 2). Mediastinal lymphadenopathy and mass were not evident in the mediastinum. In the initial laboratory tests, the patient had anemia (Hb: 11.7 g/dl and Hct: 35.4% and MCV: 81 fl), slightly increased creatinine (Cr: 1.5 mg/dl), high erythrocyte sedimentation rate (ESR 1st hour: 72 mm/h) and C‐reactive protein (CRP: 3^+^), and hematuria (blood/Hb: 3^+^and RBC: 15–20). Coagulation and liver functional tests and other biochemical tests were normal. He was admitted and tested for COVID‐19 with real‐time reverse transcription‐polymerase chain reaction (RT‐PCR). Anticoagulation was started with intravenous injection of heparin 5000 U stat continued with heparin infusion (1000 U/h). Partial thromboplastin time (PTT) was checked every 6 h to ensure it was maintained between 60 and 80 s. The result of RT‐PCR test was negative 2 days after admission.

**FIGURE 3:**
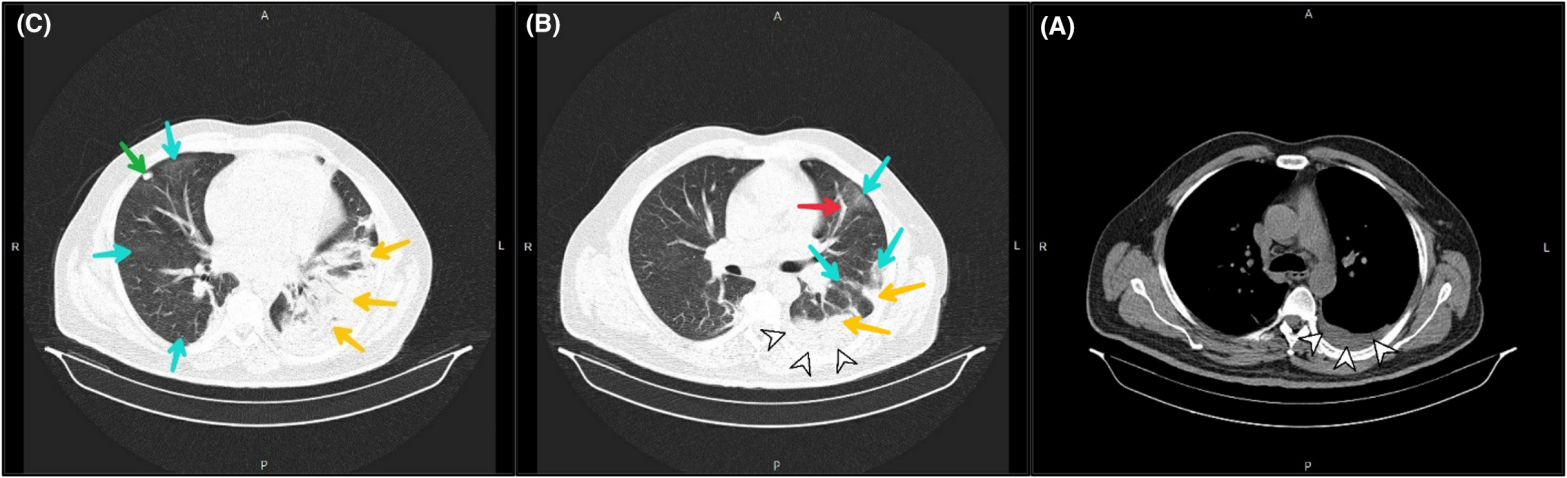
Mediastinal (A) and parenchymal (B, C) view of spiral CT scan of the patient's chest 2 weeks after admission. (A) in mediastinal view mild pleural effusion in the left lung (white arrowheads) is evident; (B) Several focal ground‐glass opacities with more intensity in the left lung (blue arrows), atelectatic band in the left lung (red arrow), alveolar consolidation areas of left lower lobe (yellow arrows), mild pleural effusion in the left hemithorax is seen in parenchymal view (white arrows); (C) Large areas of left lower lobe consolidation (yellow arrows), several focal peripheral ground‐glass opacities in the right lung (blue arrows), a nodule in the lateral segment of the middle lobe of the right lung (green arrow) is seen

**FIGURE 4:**
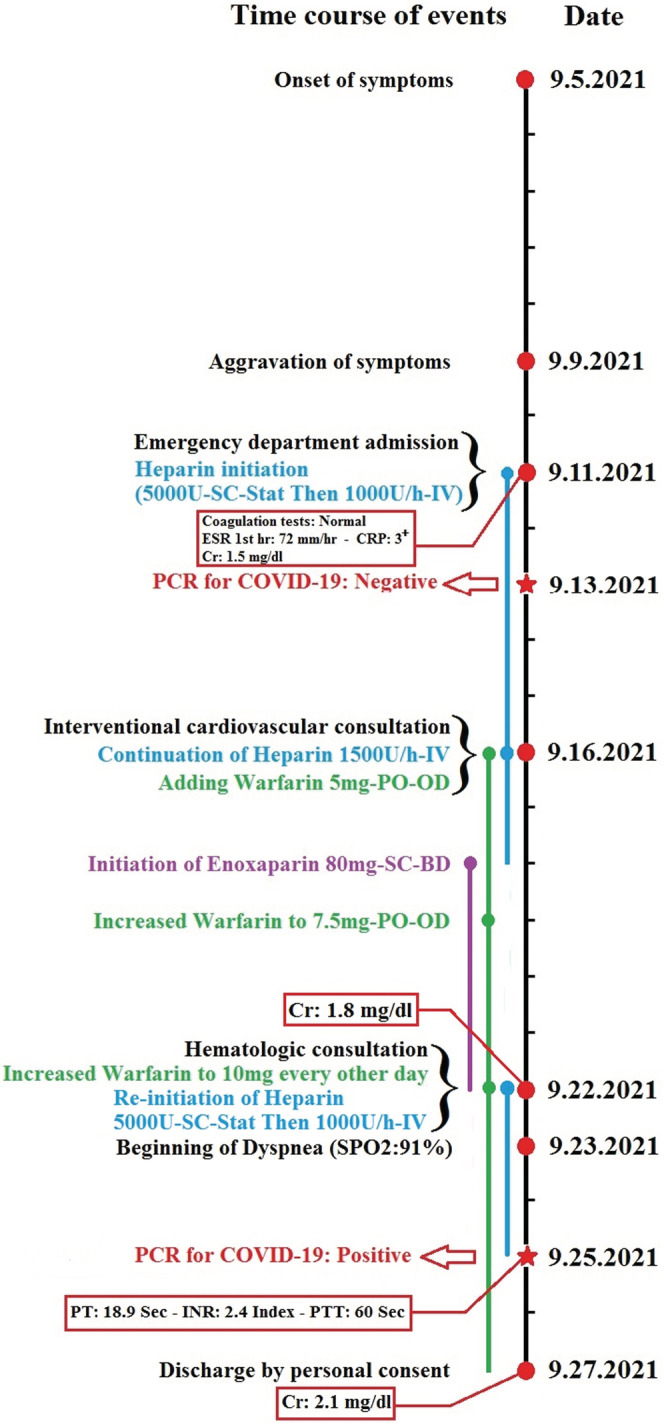
Time course of the patient's clinical events from the onset of symptoms to hospital discharge

Due to the extent of thrombosis and failure to increase PTT despite raising the heparin dose to 1500 U/h, interventional cardiovascular consultation for local thrombolytic injection was requested for the patient. However, the patient did not consent to angiography and thrombolysis. Although IVC filters are also an option when the thrombosis is free‐floating on ultrasound, consultation with the radiologist indicated that the thrombosis was completely fixed, and thus, an IVC filter placement was canceled. Considering the patient's relative resistance to heparin, 80 mg subcutaneous BD injection of enoxaparin (GFR: 67.4 ml/min based on the Cockcroft and Gault formula) and 7.5 mg warfarin tablets daily were added to his treatment protocol. Plasma creatinine increased, so enoxaparin injection was discontinued and heparin infusion was restarted. Since the prothrombin time (PT), international normalized ratio (INR), and PTT did not reach the treatment range, hematology counseling was performed and the dose of warfarin was increased to 10 mg every other day until the INR stabilized at 2–3. Anticoagulant injections were then discontinued. On the 13th day of hospitalization, the patient suffered from dyspnea, pleuritic chest pain, and dry cough and his oxygen saturation dropped to 91%. Therefore, a second PCR test and a spiral chest CT scan without contrast were requested. In addition to previous CT scan findings, the recent CT scan images showed the presence of a mild pleural effusion (at a depth of 25 mm) in the left hemithorax. Alveolar consolidation was observed in the lower lobe of the left lung, and several focal ground‐glass opacities were detected in the peripheral of both lung parenchyma with greater severity in the left lung. Viral pneumonia (COVID‐19) was suggested primarily and bacterial pneumonia in the differential diagnosis (Figure 3). After 3 days, the patient's PCR was positive, and he was transferred to the COVID‐19 ward and Cefepime Ampule 500 mg BD was started. After 15 days of admission, coagulation tests approached the target (PT: 18.9 s, INR: 2.4 Index, PTT: 60 s), the patient's heparin was discontinued, but warfarin treatment was continued. The left flank pain reduced, and the hematuria was relieved. The creatinine level increased to 2.1 mg/dl following COVID‐19 treatment. The patient left the hospital after 17 days of hospitalization with personal consent and advice to take warfarin. The time course of the patient's clinical events from the onset of symptoms to hospital discharge is shown in Figure 4. He did not cooperate and did not refer to follow‐up creatinine level after discontinuation of nephrotoxic drugs and discharge.

## 3 DISCUSSION

Several studies have reported venous thromboembolism (VTE) events, mainly PE and DVT, following COVID‐19. The incidence of VTE, especially in critically ill and mechanically ventilated patients, was estimated at 25%–30%. Other thrombotic complications including stroke, acute limb ischemia, and acute coronary syndrome have also been reported in cases of COVID‐19. The incidence rates of acute ischemic stroke and myocardial injury in hospitalized patients have been estimated at about 5% and 20%, respectively.^3^

The main role of ACE‐2 is to inactivate angiotensin 2 by its proteolytic conversion to Angiotensin (1‐7). The reduction in ACE‐2 activity after COVID‐19 infection increases the angiotensin 2 concentration and RAAS activity and decreases the Angiotensin (1‐7) concentration. Angiotensin 2 and Angiotensin (1‐7) are functionally opposite (Table 1), that is, while Angiotensin (1‐7) has beneficial effects (protective effects on organs oligopeptides), angiotensin 2 has a negative and destructive effect on the body.^6^

**TABLE 1:** Opposite effects of angiotensin 2 and Angiotensin (1‐7)

Angiotensin 2	Angiotensin (1‐7)
Vasoconstrictor	Vasodilator
Proinflammatory	Anti‐inflammatory
Induces organ fibrosis	Inhibits organ fibrosis
Prooxidative	Antioxidative
Stimulates Proliferation	Inhibits Proliferation
Prothrombotic	Anticoagulatory

Angiotensin (1‐7) prevents platelet activation and vascular endothelial damage by binding to MAS receptors on the endothelium and increasing the production of nitric oxide and prostacyclin. Through oxidative stress and various pathways, including overexpression of lectin‐like oxidized low‐density lipoprotein receptor‐1 (LOX‐1), cyclooxygenase‐2 (COX‐2), and vascular endothelial growth factor (VEGF), impaired RAAS regulation can cause endothelial damage and thus predispose the arteries to thrombotic events.^3^ In addition, endothelial damage or dysfunction contributes to the generation and activation of thrombin via the release of procoagulant factor VIII.^7^, ^8^, ^9^ Upon endothelial damage, sub‐endothelial VWF is released and acts as a molecular adhesive, binds platelets to sub‐endothelial collagen, and activates platelet aggregation and thrombosis. In severe cases, dysregulated innate immune response and widespread release of proinflammatory cytokines (cytokine storm) are involved in the pathogenesis of the disease and lead to the activation of various “immunothrombotic” pathways and blood coagulation. Complement overactivation is also observed in response to dysregulated innate immune response in COVID‐19 infection. Following an infection, neutrophils prevent the spread of microorganisms into the blood by releasing NETs.^3^ A study found the excessive NETs formation (NETosis) to be involved in various human pathologies including sepsis, vasculitis, and thrombosis.^10^ Dysregulated RAAS due to ACE‐2 inhibition in COVID‐19 can also activate mitogen‐activated protein kinases (ERK, p38, and JNK), that are early activated in the innate immune response, and thus increase the risk of thrombosis.^3^ A combination of underlying medical conditions, hospitalization, bedridden status, and COVID‐19 infection may increase the hypercoagulopathy in patients. One of the organs affected by coagulation due to COVID‐19 infection is the kidney.^11^ RVT may lead to complete or partial blockage of the renal veins. It often begins in the small intrarenal veins and subsequently spreads through the larger interlobar veins to the main renal veins and even to the IVC, causing PE. The condition rarely occurs in healthy adults and is often unilateral. About 15%–20% of patients with nephrotic syndrome may develop RVT. RVT is associated with abdominal surgery such as laparoscopic cholecystectomy, trauma, tumor invasion, or invasion of the renal vein by primary retroperitoneal diseases. The causes and mechanisms of RVT are not different from venous thrombosis in other parts of the body and involve a combination of three related factors including endothelial damage, stasis, and hypercoagulability. The clinical manifestations of RVT in adults depend on the rate, extent, and degree of venous occlusion formation. Patients may be asymptomatic or have minor nonspecific symptoms such as nausea and weakness. Sometimes, due to the presence of more nonspecific major symptoms, such as upper abdominal pain, acute flank pain, and hematuria, the condition might be mistaken with renal colic caused by renal and ureteric calculi, especially in young and healthy patients. Lack of clinical manifestations makes RVT undiagnosed and therefore less reported. However, the diagnosis of RVT is necessary considering its possible consequences, including PE and progressive renal failure associated with vascular compromise and the risks of potentially harmful treatment (including anticoagulation or thrombolysis).^12^ In addition to the risk factors mentioned in the case presentation, other risk factors for RVT include renal transplantation, Behcet's syndrome, hereditary thrombophilia, antiphospholipid syndrome, and nephrotic syndrome, which were ruled out due to history and normal test results. Malignancies, especially renal cell carcinoma can also cause RVT by invading it, but considering the normal abdominal examination, imaging and the absence of any history of weight loss, night sweats, malaise, and fever in the patient, it was excluded.^13^

The case reported in this study had a previous history of extensive DVT and a positive family history of DVT despite the negative results of hereditary thrombophilia tests. In this case, COVID‐19 could be considered as the most important risk factor for thrombotic events, including RVT, especially after the arbitrarily discontinuation of prescribed warfarin tablets.

## 4 CONCLUSION

Patients with COVID‐19 have a high risk for venous and arterial thrombotic events that may increase the risk of morbidity and mortality in these patients. Therefore, in case of hematuria and urinary symptoms, especially in patients who have other risk factors for thrombosis, the possibility of RVT should be considered and necessary measures should be taken to treat and prevent thromboembolic complications.

## AUTHOR CONTRIBUTIONS

HJR performed the medical management of the patient. MN checked and reviewed the manuscript. VS drafted and revised the manuscript. BMG Performed the imaging. AM reviewed the manuscript.

## CONFLICT OF INTEREST

The authors declare that they have no competing interest to disclose.

## CONSENT

Written informed consent was obtained from the patient to publish this report in accordance with the journal's patient consent policy.

## Data Availability

Data are available on request from the authors.
